# Binary-Weighted Neural Networks Using FeRAM Array for Low-Power AI Computing

**DOI:** 10.3390/nano15151166

**Published:** 2025-07-28

**Authors:** Seung-Myeong Cho, Jaesung Lee, Hyejin Jo, Dai Yun, Jihwan Moon, Kyeong-Sik Min

**Affiliations:** School of Electrical Engineering, Kookmin University, Seoul 02707, Republic of Korea; jo1056@kookmin.ac.kr (S.-M.C.); jejeholy@kookmin.ac.kr (J.L.); ddage0323@kookmin.ac.kr (H.J.); dai1453@kookmin.ac.kr (D.Y.); mark122@kookmin.ac.kr (J.M.)

**Keywords:** binary weighted neural networks, FeRAM array, low-power AI computing, compute in memory, processing in memory

## Abstract

Artificial intelligence (AI) has become ubiquitous in modern computing systems, from high-performance data centers to resource-constrained edge devices. As AI applications continue to expand into mobile and IoT domains, the need for energy-efficient neural network implementations has become increasingly critical. To meet this requirement of energy-efficient computing, this work presents a BWNN (binary-weighted neural network) architecture implemented using FeRAM (Ferroelectric RAM)-based synaptic arrays. By leveraging the non-volatile nature and low-power computing of FeRAM-based CIM (computing in memory), the proposed CIM architecture indicates significant reductions in both dynamic and standby power consumption. Simulation results in this paper demonstrate that scaling the ferroelectric capacitor size can reduce dynamic power by up to 6.5%, while eliminating DRAM-like refresh cycles allows standby power to drop by over 258× under typical conditions. Furthermore, the combination of binary weight quantization and in-memory computing enables energy-efficient inference without significant loss in recognition accuracy, as validated using MNIST datasets. Compared to prior CIM architectures of SRAM-CIM, DRAM-CIM, and STT-MRAM-CIM, the proposed FeRAM-CIM exhibits superior energy efficiency, achieving 230–580 TOPS/W in a 45 nm process. These results highlight the potential of FeRAM-based BWNNs as a compelling solution for edge-AI and IoT applications where energy constraints are critical.

## 1. Introduction

Artificial intelligence has become ubiquitous in modern computing systems, from high-performance data centers to resource-constrained edge devices. As AI applications continue to expand into mobile and IoT domains, the need for energy-efficient neural network implementations has become increasingly critical. While deep neural networks have demonstrated remarkable capabilities across various tasks, their computational demands often exceed the energy budgets of battery-powered edge devices [1,2,3].

Binary-weighted neural networks (BWNNs) represent a promising approach to this challenge by drastically reducing the memory and computational requirements of neural network inference. By constraining weights to binary values (+1/−1), BWNNs eliminate the need for expensive floating-point multiplications, replacing them with simple XNOR operations and bit counting. This quantization approach can reduce the amounts of memory needed drastically compared to traditional integer and floating-point operations such as INT8 and FP16 numbers. The memory reduction can lead to the low energy consumption of neural networks, enabling the easy deployment of power-constrained devices [4,5,6,7,8,9,10,11].

As one of the success cases of binary-weighted neural networks, Microsoft’s BitNet, proposed very recently, can be considered [12,13]. The BitNet model represents a groundbreaking advancement in neural network efficiency, introducing the first successful large-scale implementation of one-bit Large Language Models (LLMs). This revolutionary architecture addresses the critical challenges of deploying powerful AI models on resource-constrained devices while maintaining competitive performance with traditional full-precision models. BitNet’s performance was reported to achieve remarkable efficiency improvements across multiple metrics. Specifically, in terms of inference speed, ARM CPUs show 1.37× to 5.07× speedups with 55.4% to 70.0% energy reduction, while x86 CPUs demonstrate 2.37× to 6.17× speedups with 71.9% to 82.2% energy savings [12]. The one thing to note here is that these speedups are gained without using GPUs. Moreover, the extreme weight compression enables the running of a 100 B BitNet model on a single CPU at human reading speeds (5–7 tokens per second), making powerful AI accessible on standard single-CPU-based hardware.

Ferroelectric Random Access Memory (FeRAM) crossbar arrays offer an ideal substrate for implementing these binary neural networks in hardware. FeRAM technology leverages the persistent polarization states of ferroelectric materials to store binary information with several compelling advantages: non-volatility, high endurance, fast switching speeds, and remarkably low power consumption [14,15,16,17,18,19,20,21]. In a comparison of FeRAMs with the current DRAMs, they can offer several significant advantages over DRAM technology, particularly in terms of memory density, refresh requirements, and scaling potential. These advantages make FeRAMs promising for low-power AI computing applications in the future [14,15,16,17,18,22].

More specifically, the fundamental difference between DRAMs and FeRAMs can be found in their storage mechanism: DRAMs store information as electrical charge in capacitors that discharge over time. On the other hand, FeRAMs use the stable polarization states of ferroelectric materials. This distinction creates several key advantages for FeRAMs. First, FeRAM’s non-volatile nature eliminates the need for refresh cycles that DRAM requires approximately every 64 ms [23,24,25,26,27,28]. This results in dramatic standby power reductions in FeRAMs. This significant power saving due to the elimination of refresh operations in DRAMs can make FeRAMs very valuable for battery-powered AI computing devices. Regarding memory density, traditional DRAMs employ a 1T-1C (one transistor, one capacitor) cell structure. In contrast, FeRAMs can be made of a 1T-nF structure, where multiple ferroelectric capacitors can be stacked layer by layer. This multiple-layer stacking is crucial in achieving a higher density of three-dimensional memory crossbars than the current DRAM technology based on a two-dimensional array. In addition, DRAM scaling faces fundamental challenges as cells shrink below 15–20 nm, where DRAM’s cell capacitors struggle to store sufficient charge. Conversely, ferroelectric materials maintain their polarization properties at smaller dimensions, with demonstrated functionality at sub 10 nm [29,30,31,32,33]. Emerging ferroelectric HfO_2_ materials show particular promise for scaling to 5–7 nm nodes while maintaining reliable operation, which can hint to the possibility of FeRAM technology in a future angstrom-scale era [23,32,34].

These advantages of FeRAMs over the current DRAMs—non-volatility, potential for higher density, and a better scaling scenario—position FeRAMs as an attractive alternative to the current DRAM technology, especially for low-power AI computing, where energy efficiency is paramount. While challenges remain in manufacturing and integration, the potential benefits make FeRAM a promising technology for future memory systems, particularly in edge computing devices where power constraints are significant.

**Figure 1:**
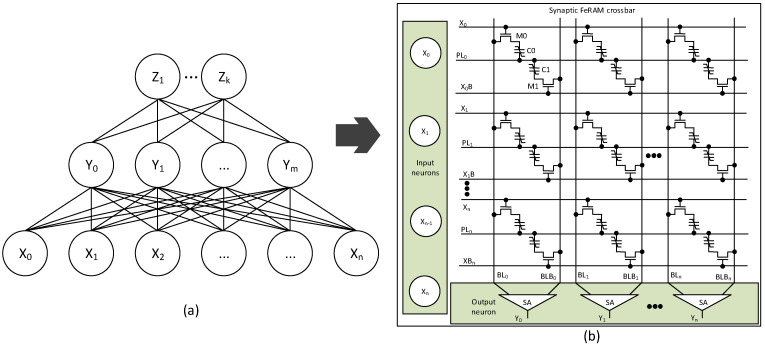
(**a**) A block diagram of artificial neural networks with input, hidden, and output neurons. (**b**) The FeRAM crossbars for implementing the neural networks.

Figure 1a shows a block diagram of normal neural networks, which is composed of input neurons (X_0_–X_n_), hidden layer neurons (Y_0_–Y_m_), and output neurons (Z_0_–Z_k_). Here, ‘n’, ‘m’, and ‘k’ are the numbers of input, hidden, and output neurons, respectively. Figure 1b shows that the neural network in Figure 1a can be physically implemented using a FeRAM-based synaptic crossbar array. In Figure 1b, each cross-point in the array integrates transistor elements (M0, M1) and capacitive elements (C0, C1) to store synaptic weights. To perform in-memory computing using the FeRAM array, first, one worldline (X0, X0B, X1, X1B, etc.) is selected according to the corresponding input data row by row. At the same time, to distinguish polarization ‘0’ and ‘1’ states, a plate line such as PL0 or PL1 is also raised from 0 V to VDD row by row. According to the stored polarization states ‘0’ or ‘1’, a bitline such as BL0 or BLB0 gains an amount of charge that can increase the bitline voltage higher than VREF or not. The voltage difference between the bitline and the inversion is sensed by the sense amplifier (SA) and produces ‘0’ or ‘1’ at the corresponding output neuron (Y0, Y1, Y2, etc.). This structure inherently supports row-serial and column-parallel execution of multiply-and-accumulate (MAC) operations, making it highly suitable for energy-efficient in-memory computing hardware for neural network inference.

The combination of binary-weighted networks and FeRAM crossbar arrays creates a powerful synergy for low-power AI computing. This approach can address both algorithmic and hardware inefficiencies in neural network implementations, delivering orders-of-magnitude improvements in energy efficiency without substantial accuracy degradation for many practical applications. As edge AI continues to evolve, this integrated approach shows tremendous promise for enabling sophisticated intelligence in energy-constrained environments, from wearable health monitors to autonomous sensors and beyond.

More specifically, for achieving energy-efficient computing, in this paper, we propose a BWNN (binary-weighted neural network) architecture implemented using FeRAM (Ferroelectric RAM)-based synaptic arrays. By combining the non-volatile nature and low-power in-memory computing of the FeRAM array, the proposed CIM (computing-in-memory) architecture of the BWNN can achieve significant reductions in both dynamic and standby power consumption. The proposed architecture is shown in Section 2 in this paper. Simulation results are shown in Section 3. Here the scaling of the ferroelectric capacitor size can reduce dynamic power more, by up to 6.5%. Moreover, eliminating DRAM-like refresh cycles allows standby power to drop by over 258× under typical conditions. Furthermore, the FeRAM-based energy-efficient inference does not show any significant loss in recognition accuracy, as validated using MNIST datasets. Finally, compared to prior CIM architectures of SRAM-CIM, DRAM-CIM, and STT-MRAM-CIM, the proposed FeRAM-BWNN can demonstrate superior energy efficiency, achieving almost 230–580 TOPS/W in a 45 nm process by circuit simulation.

## 2. Method

**Figure 2:**
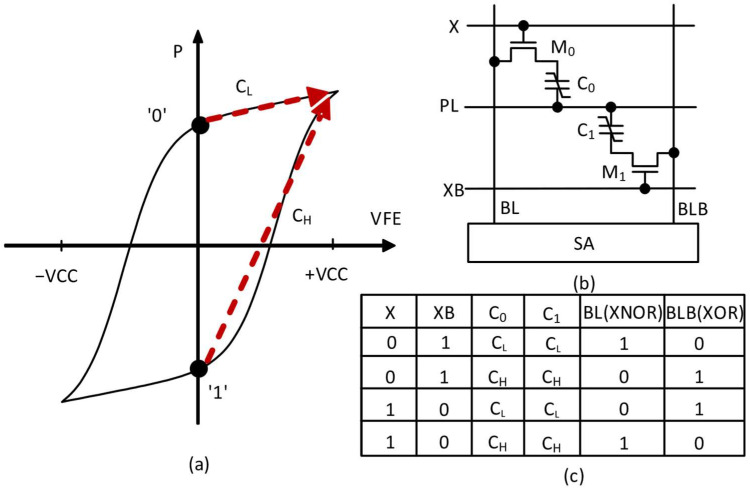
(**a**). The polarization-voltage (P-VFE) hysteresis curve of FeRAM. (**b**) The 2T-2C cell array for implementation of binary-weighted neural networks. (**c**) The truth table of XOR and XNOR operations using the FeRAM array. Here, XB is an inversion of X. C0 and C1 have the same capacitance (CL or CH). Two operands of XNOR are X and C0(=C1). Similarly, X and C0(=C1) can be two operands of XOR, too. The bitline voltages (BL and BLB) are the results of XNOR and XOR operations, respectively.

In Figure 2a, a typical polarization–voltage (P-V) characteristic curve of a ferroelectric capacitor is shown. Here ‘P’ on the y-axis means the amount of polarization and ‘VFE’ on the x-axis represents the voltage applied across the ferroelectric capacitor. The hysteresis behavior observed in Figure 2a demonstrates how two stable polarization states (‘0’ and ‘1’) can persist even when the voltage is removed, enabling non-volatile storage. In Figure 2a, red dashed lines labeled C_L_ and C_H_ represent low and high capacitance, respectively. They can be calculated by the simplified linear approximation of the ferroelectric capacitor behavior shown in Figure 2a, and are useful for circuit design and simulation.

Figure 2b shows a schematic of a 2T-2C cell composed of two transistors and two capacitors. The circuit in Figure 2b uses complementary ferroelectric capacitors (C0 and C1) connected to access transistors (M0 and M1). The wordline (X, XB) controls the access to the cells. The plate line (PL) can be used to distinguish the polarization state stored at the ferroelectric capacitor. The bitlines (BL and BLB) are used for reading and writing operations, with the sense amplifier (SA) at the bottom detecting the voltage difference between BL and BLB during the read operation.

Figure 2c represents a truth table that performs XOR and XNOR operations achievable with the circuit implementation in Figure 2b [35]. Here, XOR and XNOR mean the exclusive OR and exclusive NOR operations, respectively. The table in Figure 2c shows the relationship between input ‘X’, weight ‘W’ (potentially stored at cell capacitors such as C0 and C1), and output ‘Y’. This demonstrates how the FeRAM array can be used to perform the XOR and XNOR operations needed for realizing binary-weighted neural networks, where the synaptic weights are constrained by binary values of +1 and -1.

A more detailed explanation of the XNOR operation in Figure 2c is as follows: Here, XB is an inversion of X. C0 and C1 have the same capacitance (CL or CH). Two operands of the XNOR Boolean operator are regarded as X and C0(=C1). Similarly, X and C0(=C1) can be two operands of XOR, too. The bitline voltages (BL and BLB) are the results of XNOR and XOR operations, respectively, as shown in Figure 2c. If the resulting BL voltage from the XNOR operation is VDD, it is regarded as logic ‘1’. If the BL voltage is 0 V, it is regarded as logic ‘0’. When X = 0 and XB = 1, if the plate line (PL) is driven to be high, the voltage on BLB is raised in proportion to C1. If C1 is as low as CL, BL and BLB become logic ‘1’ and ‘0’, respectively. On the other hand, if C1 is as high as CH, BL and BLB become logic ‘0’ and ‘1’, respectively. As mentioned earlier, C0 is always the same as C1. Unlike C0 and C1, X and XB have a complementary relationship, as shown in Figure 2c. This means that X is an inversion of XB. Considering X and CL(=CH), when X and C0 are both low, BL becomes high. In contrast, when X = 0 and C0 is high, BL becomes logic ‘0’. Similarly, when X = 1 and XB = 0, we can find the same XNOR results as shown in Figure 2c. One more comment to make here is that the XNOR and XOR operations that can be found on BL and BLB, respectively, in Figure 2b are based on the read operation of the FeRAM array. By this means, the Boolean operations such as XNOR and XOR can be performed with low power consumption, because the memory access and logic operation can be combined together and performed simultaneously.

**Figure 3:**
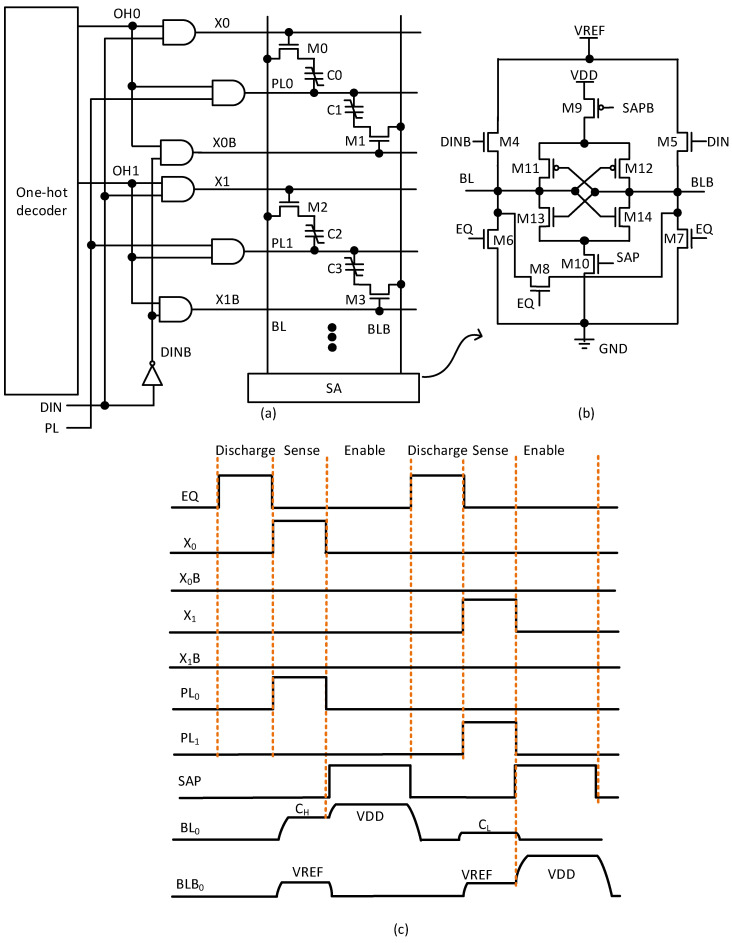
(**a**) The schematic of FeRAM array and one-hot decoder for implementing binary-weighted neural networks. (**b**) The schematic of the sense amplifier (SA) circuit. (**c**) The timing diagram of SA operation.

Figure 3a presents a schematic of the FeRAM array and one-hot decoder for implementing binary-weighted neural networks. In this figure, the one-hot decoder is used to activate wordlines of the FeRAM array one by one according to the predetermined order. From the top row to the bottom one, the one-hot decoder generates enabling signals (OH0, OH1) row by row. The generated signals (OH0, OH1) are combined with input data (DIN) to decide whether X or XB is activated. The one-hot decoder enables efficient addressing of the FeRAM array when performing neural network operations.

The operation of the FeRAM array in Figure 3a can be explained in more detail. The array features multiple 2T-2C FeRAM cells arranged in rows. Each cell contains complementary ferroelectric capacitors such as C0/C1 and C2/C3 paired with access transistors of M0/M1 and M2/M3. This differential structure operates based on the binary input data. When DIN is 0, C1 and M1, which are connected to BLB, are activated. Conversely, when the input is 1, C0 and M0, which are connected to BL, become active. The wordlines (X0, X1) and their complements (X0B, X1B) are driven by logic gates that combine the one-hot signals (OH0, OH1) with the input data (DIN). Parallel plate lines (PL0, PL1) are applied to drive the ferroelectric capacitors during the read and write operations. This arrangement allows for selective activation of specific cells during computation. The bitlines (BL, BLB) run vertically and connect to the SA at the bottom of the array. These lines carry the differential signals generated by the ferroelectric capacitors when accessed, forming the data path for neural network operations.

Figure 3b details a sense amplifier circuit that detects small voltage differences between BL and BLB. The sense amplifier employs a cross-coupled latch structure (M11–M14) that amplifies the small differential voltage to a full VDD voltage. Figure 3b includes discharge and equalization circuitry (M6–M8) controlled by an EQ signal. When the EQ is high, the bitlines are equalized to a ground voltage before a read operation, establishing a known starting point for accurate sensing. Control signals SAP and SAPB activate the amplification process. When the SAP is high and the SAPB is low, the latch is enabled and amplifies the bitline voltage difference. This design ensures high-speed operation and reliable data detection even with the small signals delivered by the ferroelectric cells. The transistors of M4 and M5 controlled by DIN and DINB provide the write path, allowing new data to be written to the selected memory cell. Also, on the read operation, depending on the data at the input, the DIN turns on M5 to make BLB go to VREF, when the input is 1. Similarly, BL goes to VREF when the input is 0. The entire circuit is powered by VDD and GND, with careful signal levels to ensure proper polarization of the ferroelectric capacitors.

Figure 3c shows a precise timing sequence for FeRAM array operation, which is critical for correct binary neural network computation. The diagram tracks multiple control signals across a complete read–write cycle as follows:

1. The sequence begins with EQ high, equalizing the bitline and its inverted version. At the same time, the bitlines are discharged to the ground voltage to prepare for a read operation.
2. X0 is then activated when DIN = 1, selecting the first row of the memory array. In contrast, if DIN = 0, XB0 is enabled instead of X0. At the same time, PL0 is activated to read the stored polarization state.
3. SAP is then triggered, activating the sense amplifier to detect and amplify the small voltage difference between the bitline and its inversion.
4. The resulting voltage levels on BL0 and BLB0 diverge based on the stored data, with one rising above VREF and the other falling below it.
5. After the first operation is completed, a second operation occurs with X1 and PL1 activating the next row.

As explained earlier, the timing diagram in Figure 3c demonstrates how the array can sequentially access different memory cells, which is essential for implementing binary-weighted neural network computations where multiple weights must be accessed and processed in sequence.

**Figure 4:**
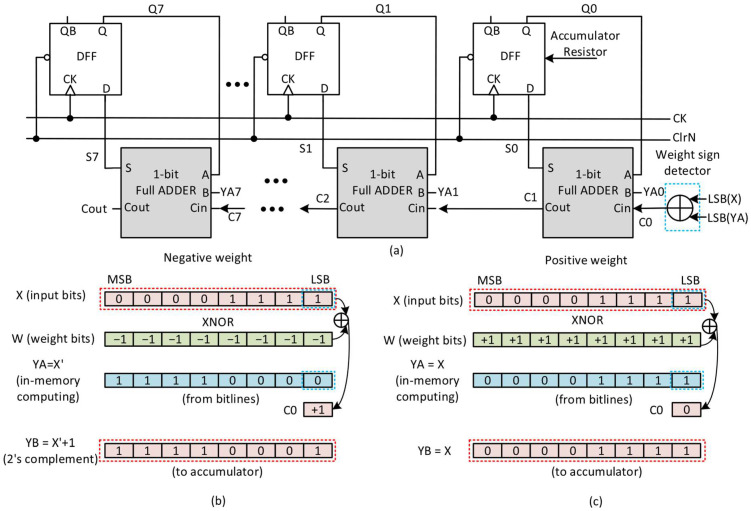
(**a**) The block diagram of the accumulator used at hidden and output neurons based on a simple ripple carry adder architecture. (**b**) The operation of X and W for the accumulator when the binary weight is −1. (**c**) The operation of X and W for the accumulator when the binary weight is +1.

Figure 4a presents a block diagram of the accumulator used at hidden and output neurons based on a simple ripple carry adder architecture. The block diagram shown in Figure 4a has multiple 1-bit full adders connected serially to perform the carry-propagate addition from LSB to MSB. Here, LSB and MSB represent the Least significant bit and Most significant bit, respectively. Each one-bit full adder receives its inputs from D flip-flop (Qn), bitline (Yn), and carry-in (Cn), producing a sum (Sn) and carry-out (Cout). The outputs of these one-bit full adders are latched using the D flip-flops (DFFs), which accumulate the intermediate results over multiple cycles. A global clock (CK) and clear signal (ClrN) manage the timing and reset behavior of the accumulator. Additionally, a ripple carry chain (C0 to C7) propagates the carry bits through the adder stages, enabling multi-bit-precision accumulation. A weight sign detector shown in the right–bottom corner processes the sign of weight to ensure the correct summation based on the weight polarity. Here, if the sign of weight is negative, C0 becomes 1. On the contrary, if the weight is positive, C0 is 0. The weight sign detector calculates C0 to convert a negative number to its two’s complementary format. For a positive number, it keeps its original bits, not being converted to the two’s complement.

Figure 4b demonstrates the case of negative weight. Here, X and W mean input bits and weight bits, respectively. YA means the result of in-memory computing of the FeRAM array. Here, YA can be sensed from the bitline. As explained earlier, the FeRAM array can perform XNOR operation. If the W = −1, the XNOR operation’s result for YA is the inversion of X, which is denoted as X’. To detect the sign of weight, the XOR operation is performed for the LSB of X and LSB of YA, as shown in Figure 4b. If the XOR result is 1, it means that the sign of weight is negative. In the context of this minus sign, C0 becomes 1 and is delivered to carry-in to take into account the two’s complement. In Figure 4b, YB means the final result of the multiplying operation, which can be expressed with YB = X’ + 1.

Similarly, Figure 4c demonstrates how a positive weight is handled in the accumulation process. For a positive, the XNOR result is the same as the input X, as expressed with YA = X. To detect the sign of weight, the XOR operation is performed for the LSB of X and LSB of YA, as shown in Figure 4c. If the XOR result is 0, it means that the sign of weight is positive. To consider this positive sign, C0 becomes 0 and is delivered to carry-in. In this case, finally, the result of the multiplying operation can be expressed with YB = X. This simple architecture, based on the FeRAM array, SA, and accumulator, can be extended from fully connected DNNs to convolutional neural networks (CNNs), enabling inference on a wide range of datasets—from 2D grayscale images such as MNIST to color image datasets like CIFAR-10.

## 3. Results

**Figure 5:**
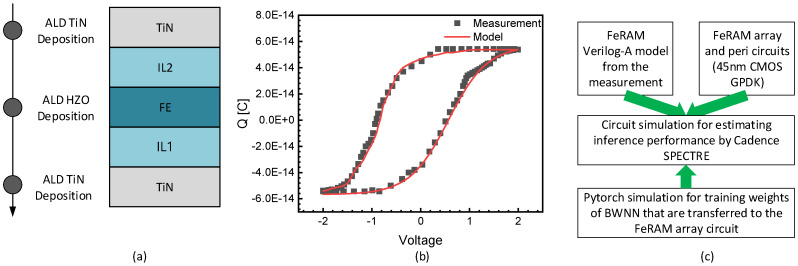
(**a**) A cross-sectional view of the ferroelectric capacitor measured, data from reference [36]. (**b**) The voltage–charge (V–Q) characteristics of the ferroelectric capacitor with the measurement and Verilog-A model. The modeling is performed using the Verilog-A language. (**c**) A block diagram of the circuit simulation performed in this work. The FeRAM Verilog-A model is developed using the Verilog-A language, and the model is integrated with the FeRAM array and peripheral circuits designed using the CADENCE 45 nm CMOS GPDK. The circuit simulation is performed by the CADENCE 6 SPECTRE tool, which can calculate both the CMOP circuits and Verilog-A model. The circuit simulation is for estimating the inference performance of BWNN. The pytorch simulation is needed for training weights of the BWNN that are transferred to the FeRAM array.

Figure 5a shows a cross-sectional view of the ferroelectric capacitor measured and modeled in this paper [36]. The ferroelectric device is made of Pr-enhanced Hf_0_._5_Zr_0_._5_O_2_ (HZO) film, where its low-voltage operation and high-density potential can make this device suitable for energy-efficient AI computing [36]. The developed ferroelectric device has a stacking architecture of high-endurance metal–ferroelectric–metal (MFM) films. Here the ferroelectric film undergoes interlayer (IL) curing treatment. To form the bottom and top electrodes of the FeRAM capacitor, novel materials such as α-TiN are used. The measured remnant polarization (2Pr) can be as large as 54 μC/cm^2^ and the measured endurance is observed to exceed 10^12^ cycles at 85 °C for the FeRAM device measured in this paper. This FeRAM array can be integrated vertically with CMOS devices for forming a 1T-1F or 1T-nF architecture. For the operation of the 1T-nF array, plate lines to drive FeRAM cells should be separated layer by layer. On the other hand, the 1T-1F array has only one common plate line without multiple-layer stacking. By this means, the sensing voltage margin of the 1T-1F array can be better than the 1T-nF array even when VDD is very low.

One more thing to note here is that FeRAM devices are non-volatile so that they do not need to be refreshed periodically, unlike DRAMs. The DRAM cell stores the information at a storage capacitor as an amount of charge. The stored charge in the DRAM cell should be refreshed periodically before too much of the charge is lost. This periodic refresh operation of the DRAM array consumes a large amount of switching power even during the standby mode. In contrast, the FeRAM array can maintain the information as a polarization state, keeping the state for a long time. This advantage highlights FeRAM’s strong potential for low power consumption during the standby mode.

Figure 5b shows the voltage–charge (V–Q) characteristics of the ferroelectric capacitor with the measurement and its Verilog-A simulation model. The modeling of the V-Q curve is performed using the Verilog-A language, which can be simulated with commercial CAD tools such as CADENCE SPECTRE. In Figure 5b, black square symbols represent the measurement, while red lines indicate the Verilog-A simulation model. The modeled curves are in good agreement with the measurement, as shown in Figure 5b. The model can successfully reproduce the typical hysteresis behavior of the ferroelectric capacitor, including the saturation at high voltages, the sharp transition around the coercive voltage, and the remnant polarization near zero volt. These V–Q responses are critical for accurately simulating FeRAM cells, as they govern charge accumulation and retention under different bias conditions. The behavioral model captures the nonlinear polarization dynamics, enabling accurate prediction of write/read characteristics of the FeRAM array.

For explaining the Verilog-A simulation model in detail, the hysteresis behavior of the FeRAM capacitor can be described using Equations (1)–(3), as shown below.

(1) $$q(v(t))=Q_{H}\;\cdot tanh(a\cdot V-b),\;\mathrm{for}\;C_{H}$$

First, Equation (1) defines the stored charge, *q*(*v*(*t*)), when the polarization state is ‘1’. In this case, the ferroelectric capacitance is as large as C_H_. Equation (1) is modeled using a scaled hyperbolic tangent function with the magnitude parameter of *Q_H_*, the slope factor of ‘a’, and the offset voltage of ‘b’. The offset corresponds to the positive coercive voltage.

(2) $$q(v(t))=Q_{L}\;\cdot tanh(c\cdot V+d),\;\mathrm{for}\;C_{L}$$

Similarly, Equation (2) represents the charge behavior for the polarization state of ‘0’, where the tanh function is shifted by the offset d, and the parameter *c* is used as a slope factor of Equation (2). The saturation charge is denoted as Q_L_, which can be similar to or different from Q_H_, depending on asymmetry in device characteristics.

(3) $$i(t)=\frac{dq(v(t))}{dt}$$

Equation (3) describes the current *i*(*t*) as the time derivative of the stored charge *q*(*v*). By applying the chain rule, the current is expressed as the time derivative of a scaled tanh function, where the voltage waveform and its rate of change *dV*/*dt* directly influence the current response. The model parameters used in Equation (1) are Q_H_ = 5.67 × 10^−14^, a = 1.26, and b = −0.72, while those used in Equation (2) are Q_L_ = 5.5 × 10^−14^, c = 2.29, and d = 1.78. The Verilog-A model in Figure 5b is calculated with the parameters shown above, and the measurement data in Figure 5b were obtained from reference [36].

Figure 5c shows a block diagram of the circuit simulation performed in this work. The FeRAM Verilog-A model in Figure 5b is developed using the Verilog-A language as mentioned earlier. This model is integrated with the FeRAM array and peripheral circuits designed using the CADENCE 45 nm CMOS GPDK (Generic Process Development Kit, Cadence Design Systems, Inc. San Jose, CA, USA). The circuit simulation is performed by the CADENCE SPECTRE tool, which can calculate both the CMOS circuits and Verilog-A model in this paper. The circuit simulation is for estimating the inference performance of the BWNN. The pytorch simulation is needed for training weights of the BWNN that are transferred to the FeRAM array.

**Figure 6:**
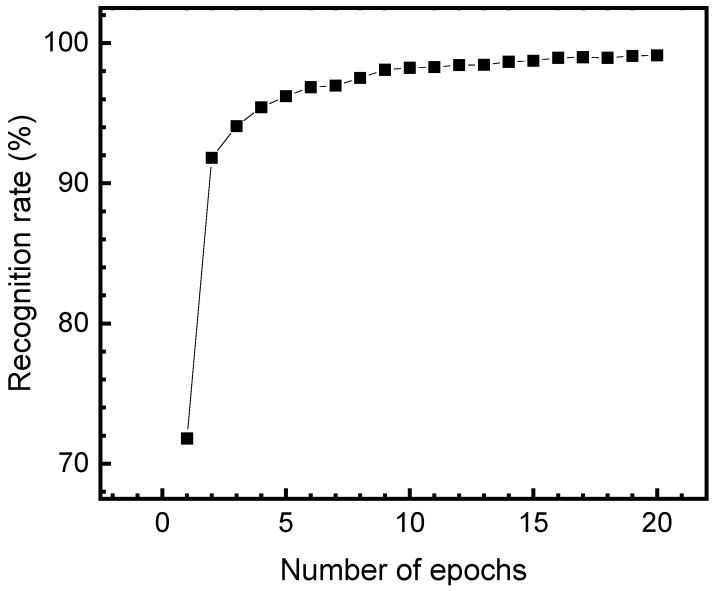
The improvement in recognition rate with an increasing number of epochs for the binary-weighted network using the FeRAM array. The recognition rate is evaluated by the MNIST dataset.

Figure 6 shows the training performance of the binary-weighted neural network evaluated on the MNIST dataset. The network consists of four fully connected layers with sizes 784–256–64–10. The input neurons are quantized to six bits, while hidden and output neurons are represented using eight-bit precision. All weights in the network are constrained to binary values (+1/−1). As shown in Figure 6, the recognition rate of the binary-weighted network evaluated on the MNIST dataset increases with the number of training epochs. The recognition rate improves rapidly during the initial epochs, increasing from approximately 70% to over 90% within the first two epochs. The accuracy continues to improve gradually and saturates around 99% after 15 epochs, indicating effective convergence of the training process. Here, the training is performed using pytorch 2.5.1 software as indicated in Figure 6. The inference of the BWNN implemented by the FeRAM array is tested by circuit simulation.

One more thing to note is that we compared our simulation results of the binary-weighted neural network with previous works [6,37]. The neural network’s recognition rate simulated in this work is very comparable to the previous works, which reported an accuracy as high as 99 ± 0.5% [6,37]. One thing to note here is that the novelty of this work is not in the recognition accuracy but in the low energy consumption of in-memory FeRAM computing. As highlighted earlier, the binary-weighted neural networks are implemented by the FeRAM array in this work. By doing so, we could achieve very energy-efficient computing of binary-weighted neural networks.

**Figure 7:**
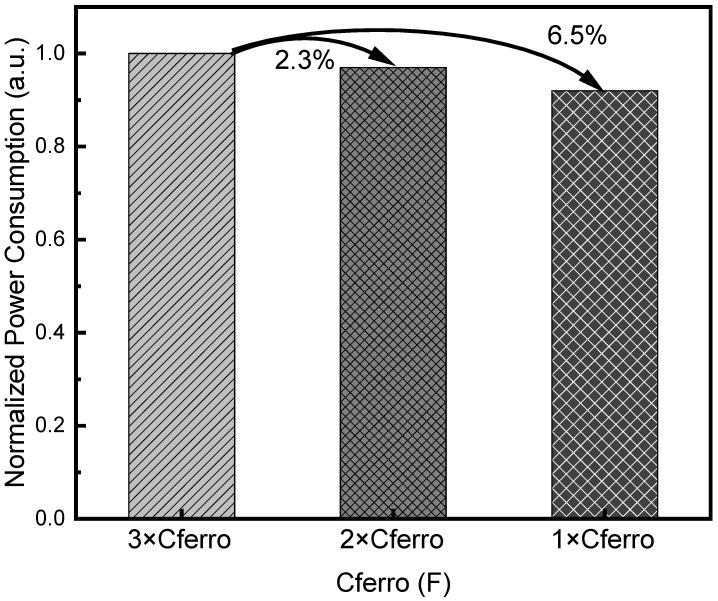
The power consumption for different ferroelectric capacitor sizes in the FeRAM array. The first, second, and third columns represent Cferro = 3×, Cferro = 2×, and Cferro = 1×, respectively.

Figure 7 presents the effect of scaling ferroelectric capacitor (Cferro) size on overall power consumption. In Figure 7, the first column represents a ferroelectric capacitor size as large as 3×. The second and third columns represent ferroelectric capacitor sizes as large as 2× and 1×, respectively. The ferroelectric capacitance size of 3× is obtained from the measurement, where CH = 90 fF and CL = 8 fF were observed from the FeRAM cell’s hysteresis behavior [36]. As the FeRAM technology node advances further, the ferroelectric capacitance size is expected to be scaled down. To estimate how much the dynamic power consumption of the FeRAM array can be reduced by the scaling of ferroelectric capacitance, Cferro = 3× and Cferro = 1× are considered in Figure 7. Cferro = 2× means that the capacitance is 2/3 of Cferro = 3×. Similarly, Cferro = 1× means that the capacitance is 1/3 of Cferro = 3×. When comparing Cferro = 3× and Cferro = 1×, the power consumption of the FeRAM array can be reduced by 6.5%. This simulation result highlights the importance of scaling of Cferro, which can be performed by decreasing the ferroelectric capacitance size. By this means, the dynamic power in the FeRAM array can be reduced further with the FeRAM cell’s scaling. For accurately simulating the power consumption of the FeRAM-based neural network, the FeRAM array and all the peripheral circuits such as the one-hot decoder and accumulator are considered in this simulation. As mentioned in Figure 5c, the simulation tool of CADENCE SPECTRE was used with the CADENCE 45 nm GPDK. Moreover, the FeRAM’s hysteresis behavior was included in the circuit simulation using the Verilog-A model mentioned earlier in Figure 5b.

**Figure 8:**
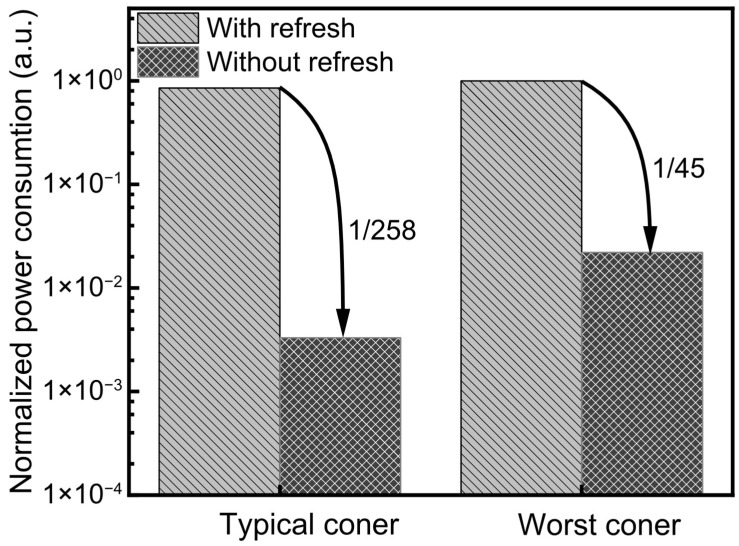
The comparison of standby power between DRAM-based and FeRAM-based CIM architectures under typical- and worst-corner conditions. The typical corner means conditions of TT and 27 °C. Under the worst corner, the conditions are FF and 120 °C. Here the worst corner means the condition is the worst in terms of leakage power during the standby mode.

Figure 8 presents a comparison of standby power consumption of the binary-weighted networks between DRAM-based and FeRAM-based architectures. As illustrated, the DRAM-based array exhibits significantly higher standby power due to the need for a periodic refresh operation to retain the stored data. These refresh cycles are inherent to DRAM’s volatile nature and result in continuous energy expenditure even when the system is idle during the standby mode. In contrast, if the neural network is implemented using the FeRAM-based architecture, it shows a substantial reduction in the standby power. This is attributed to the non-volatile nature of FeRAM, which retains data using the polarization state of ferroelectric materials without requiring refresh. During the standby mode, FeRAM incurs only minimal sub-threshold leakage current, as no active switching or refresh is needed. This dramatically lowers the overall static power consumption.

The effectiveness of this refresh-free operation is further highlighted in Figure 8, which shows normalized standby power under both typical (TT, 27 °C) and worst-case (FF, 120 °C) corner conditions for DRAM-based and FeRAM-based CIM architectures. Here, ‘CIM’ stands for computing-in-memory. As shown, the FeRAM-based architecture achieves a dramatic reduction in standby power due to its refresh-free nature. At the typical corner, the FeRAM system without refresh consumes only 1/258 of the standby power compared to the DRAM system with refresh. Even under worst-case conditions, it still achieves a 45× reduction, highlighting the robustness of FeRAM’s low-power characteristics across varying PVT conditions. Here, ‘PVT’ means process–voltage–temperature.

The elimination of refresh operations and reduction in leakage to sub-threshold levels make FeRAM highly advantageous for energy-constrained applications such as mobile devices, edge-AI systems, and IoT nodes. These results confirm that FeRAM-based CIM architectures offer not only functional benefits but also superior energy efficiency and scalability for future low-power intelligent hardware.

Table 1 presents a comparative analysis of recent computing-in-memory (CIM) architectures, including a digital SRAM-based CIM, an STT-MRAM-based CIM, a DRAM-based PIM architecture, and the proposed FeRAM-based design [38,39,40]. The comparison spans key design metrics such as fabrication technology, operating voltage, bit precision, and energy efficiency.

The digital SRAM-based CIM was implemented in a 5 nm FinFET process and was observed to achieve up to 254 TOPS/W and 221 TOPS/mm^2^, leveraging a 12 T bit cell for simultaneous MAC and write operations, as well as support for dynamic voltage–frequency scaling [38]. In contrast, the STT-MRAM-based CIM demonstrated 129.8 TOPS/W in a 22 nm process, highlighting its energy-efficient characteristics alongside non-volatility, making it favorable for advanced edge-AI chips [39]. The DRAM-PIM architecture, fabricated in a 28 nm process, achieved 19.36 TOPS/W, offering a dense memory-integrated transformer core for hybrid sparse–dense AI computations [40]. However, its large DRAM cell size and high refresh energy overhead make it less energy-efficient compared to the other CIM counterparts.

In Table 1, the proposed FeRAM-based design seems to surpass many of these architectures such as SRAM-CIM, MRAM-CIM, and DRAM-CIM in terms of computing power efficiency. Specifically, at the lowest VDD = 0.6 V, the proposed FeRAM-based neural network shows as high as 580 TOPS/W, which is much higher than that of the SRAM-CIM. One more thing to note here is that the non-volatility of FeRAM can reduce the standby power more drastically than DRAM-PIM, as explained in Figure 8. In a comparison of the sub-threshold leakage between the SRAM-CIM and FeRAM-CIM, the FeRAM-CIM is estimated to have much lower leakage than the SRAM-CIM. This is because the FeRAM-CIM can be powered down completely during the standby mode, while the SRAM-CIM should not be in order to keep the cell data. The non-volatility is a big advantage of FeRAM over the SRAM-CIM and DRAM-CIM, whose memory cells are volatile. In a comparison of the MRAM-CIM and FeRAM-CIM, the MRAM-CIM consumes more energy than FeRAM-CIM, because the MRAM’s small on–off resistance ratio requires more energy consumption by MRAM’s sense amplifier. Due to the FeRAM’s inherent non-volatility and competitive energy efficiency, the FeRAM-CIM can be regarded as a potential candidate for a scalable and energy-conscious memory solution for edge-AI applications.

One more thing to comment on here is the cell area issue that leads to the area efficiency of the CIM architecture, defined in TOPS/mm^2^. As is well known, the SRAM-CIM is the worst in terms of area efficiency because the SRAM’s cell composed of 6T-12T occupies a much larger area than DRAM, MRAM, and FeRAM. The commercial DRAM cell can be scaled down to a small area, but the DRAM-CIM fabricated by a CMOS logic foundry cannot be scaled down to as small as the commercial DRAM fabricated by a DRAM-oriented IDM (Integrated Device Manufacturer). MRAM can be much smaller than SRAM in terms of cell area. However, FeRAM with 1T-nF can have a dense cell array, where one access transistor can be shared by multiple ferroelectric capacitors. The ferroelectric capacitor array can be stacked layer by layer by sharing one wordline transistor among many FeRAM cells. The 1T-nF FeRAM architecture can result in a high area efficiency of FeRAM-CIM.

Let us add some practical comments on the area efficiency to this paper. A comparison of the values of TOPS/mm^2^ directly among SRAM- CIM, MRAM- CIM, DRAM- CIM, and FeRAM-based CIM architectures is not available in this paper, because the various CIM architectures reported in Table 1 were fabricated using different technology nodes. However, their cell sizes can be roughly compared with each other, as indicated in Table 1. For the SRAM array, it has been well known that the cell size of 6T-12T can be estimated to be as large as 100–200 F^2^ [41]. Here, ‘F’ means a minimum feature dimension defined by a photolithographic limit. The MRAM’s cell size was reported to be around 20–40 F^2^, and it is expected to be very difficult to stack the MRAM array layer by layer [42]. The DRAM’s cell size was reported to be 6 F^2^ for the open-bitline architecture and 8 F^2^ for the folded-bitline architecture [43]. The DRAM’s cell size seems much smaller than MRAM’s. However, the problem of DRAM is that the DRAM array is very difficult integrate with the CMOS logic process, because the DRAM cell array needs some special process technology for controlling the DRAM’s refresh characteristic. The FeRAM’s cell size can be 6–30 F^2^ for the 1T-1F architecture, indicating a wide range of cell sizes, which means that the FeRAM process is not mature yet. However, the FeRAM array can be stacked layer by layer, which is denoted as the 1T-nF architecture and can be fabricated in much smaller dimensions than the 1T-1F cell architecture [44]. Here, 1T-1F means one transistor and one ferroelectric capacitor. 1T-nF means one transistor with multiple ferroelectric capacitors.

One thing to note finally is that the FeRAM array has suffered immaturity of its process technology until now. Based on the limitation of process and device technology, FeRAM’s endurance can be lower than that of DRAM and SRAM, which actually have endless endurance [36]. Moreover, the variability and retention issues of the FeRAM array also need to be improved further in the future [45].

## 4. Conclusions

This work presents a binary-weighted neural network (BWNN) architecture implemented using FeRAM-based synaptic arrays for low-power AI computing. By leveraging the non-volatile nature and fast-switching characteristics of ferroelectric memory, the proposed system achieves significant reductions in both dynamic and standby power consumption. Simulation results demonstrate that scaling the ferroelectric capacitor size can reduce dynamic power by up to 6.5%, while eliminating DRAM-like refresh cycles allows standby power to drop by over 258× under typical conditions. Furthermore, the combination of binary quantization and in-memory computing enables energy-efficient inference without significant loss in recognition accuracy, as validated using MNIST datasets. Compared to prior CIM architectures, the proposed design exhibits superior energy efficiency, achieving 230–580 TOPS/W in a 45 nm process. These results highlight the potential of FeRAM-based BWNNs as a compelling solution for edge-AI and IoT applications where energy constraints are critical.

## Figures and Tables

**Table 1:** Comparison of SRAM-CIM, MRAM-CIM, DRAM-CIM, and FeRAM-CIM (this work) in terms of fabrication technology, supply voltage, input/weight/output precision, computing energy efficiency, and area efficiency. For the FeRAM-CIM, the cell size can be small and the layers can be stacked. By this means, FeRAM-CIM with the 1T-nF architecture seems to achieve better area efficiency than the others.

	SRAM-CIM [38]	MRAM-CIM [39]	DRAM-CIM [40]	FeRAM-CIM (This Work)
Fabrication Technology	5 nm	28 nm	28 nm	45 nm
Power Supply(V)	0.5–0.9	1	0.85–1	0.6–1
Input Bits	4	1	4	6
Weight Bits	4	1	4	1
Output Bits	14	8	4	8
Energy Efficiency (TOPS/W)	63(8b)–254(4b)	129.83	4.8–19.4	230–580
Area Efficiency (TOPS/mm^2^)	Poor (cell size: 100~200 F^2^)	Moderate (cell size: 20–40 F^2^)	Moderate (cell size of DRAM-oriented IDM process: 6–8 F^2^)	Can be good for 1T-nF (1T-1F: 6–30 F^2^, 1T-nF can be much smaller than 1T-1F)

## Data Availability

Dataset available on request from the authors.
